# Impact of *MMP2* rs243849 and rs14070 genetic polymorphisms on the ischemic stroke susceptibility in Chinese Shaanxi population

**DOI:** 10.3389/fneur.2022.931437

**Published:** 2022-07-25

**Authors:** Shilin Li, Shiyao Yang, Xiaobo Zhang, Yu Zhang, Jie Zhang, Xiao Zhang, Weiping Li, Xiaochen Niu, Wenzhen Shi, Gejuan Zhang, Mingze Chang, Ye Tian

**Affiliations:** ^1^Department of Neurology, The Affiliated Hospital of Northwest University, Xi'an No.3 Hospital, Xi'an, China; ^2^The College of Life Sciences, Northwest University, Xi'an, China; ^3^Xi'an Key Laboratory of Cardiovascular and Cerebrovascular Diseases, Medical Research Center, The Affiliated Hospital of Northwest University, Xi'an No.3 Hospital, Xi'an, China

**Keywords:** *MMP2*, susceptibility, single nucleotide polymorphism (SNP), rs243849, rs14070, ischemic stroke (IS)

## Abstract

**Background:**

Ischemic stroke (IS) is a complex neurological disease affected by genetics and environment. Matrix metalloproteinase-2 (*MMP2*) is involved in extracellular matrix (ECM) degradation, inflammation and angiogenesis to regulate the development and recovery of IS.

**Purposes:**

The aim of this study was to explore the association of rs1053605, rs243849 and rs14070 in *MMP2* with the risk of IS in Chinese Shaanxi population.

**Methods:**

In this study, 677 IS patients and 681 normal controls were recruited. Rs1053605, rs243849 and rs14070 in *MMP2* were genotyped. Logistic regression analysis was applied to evaluate the association of rs1053605, rs243849 and rs14070 in *MMP2* with IS susceptibility and the association of environmental factors with *MMP2* genetic susceptibility to IS.

**Results:**

The results of the overall analysis demonstrated that rs14070 in *MMP2* significantly reduced the risk of IS in Chinese Shaanxi population (OR = 0.767, 95% CI = 0.619–0.952, *P* = 0.016). Subgroup analysis illustrated that rs243849 in *MMP2* evidently increased the risk of IS among drinkers, while rs14070 in *MMP2* apparently reduced IS susceptibility among females, participants with aged >55, smokers and drinkers.

**Conclusions:**

Collectively, rs243849 and rs14070 in *MMP2* were significantly associated with the risk of IS in Chinese Shaanxi population, and the effect of *MMP2* to IS may be associated with its genetic susceptibility.

## Introduction

Stroke is caused by cerebrovascular disorders, including ischemic stroke (IS) and hemorrhagic stroke (HS), of which IS accounts for 70 to 90% (1, 2). High morbidity, high recurrence, high mortality, high disability and low cure rate are the main characteristics of IS (3). As a multifactorial complex neurological disorder, stroke involves clinical, environmental and genetic factors (4). Advanced age, smoking, drinking, hypertension and diabetes are the risk factors to IS (5, 6). Study indicated that the incidence of IS after the age of 40 increased evidently with age, and the susceptibility of IS in male was obviously higher than that in female in the same age group (7). There was a significant dose effect between the numbers of daily cigarettes smoked and IS in young men (8). However, clinical and environmental factors do not adequately explain differences in IS disease progression (9). Epidemiological analysis shows genetic factors play a crucial role in IS susceptibility (2, 6), accounting for 50% (10). Therefore, there is an urgent need to study the genetic variants associated with the occurrence of IS (11).

Vascular inflammation is a key factor in IS (11). Inflammation can not only promote thrombus formation and improve the stability of thrombus, but also damage the blood-brain barrier (BBB) (12). Matrix metalloproteinases (MMPs), as a class of proteolytic zinc-dependent enzymes, can regulate cytokines (13), angiogenesis (10), extracellular matrix (ECM) degradation (14), and cause BBB disorders (4), which affect the pathogenesis of vascular inflammation, stroke and atherosclerosis (11). It has been reported that the MMPs associated with IS were mainly concentrated in *MMP2* and *MMP9* (15). *MMP2* also regulates angiogenesis, vascular inflammation, ECM degradation, and BBB disruption, which are critical to IS occurrence, progression, and recovery (13). Genetic polymorphisms of *MMP2* affect its transcription and expression (10), a preliminary study found that rs1132896 and rs243849 of *MMP2* evidently reduced the risk of IS in southern Chinese populations (9). However, genetic variation in *MMP2* were not notably associated with the risk of IS in the Han Hakka populations (16). Due to the differences in the risk correlation between *MMP2* and IS in different populations, the study of *MMP2* polymorphisms and IS susceptibility in different populations needs to be explored extensively.

The purpose of this study was to investigate the effect of *MMP2* polymorphisms on the occurrence of IS in Chinese Shaanxi population. Three SNPs (rs1053605, rs243849 and rs14070) in *MMP2* were selected and logistic regression analysis with OR and 95% CI values was used to evaluate the association between *MMP2* polymorphisms and IS susceptibility. Our research results will hopefully provide valuable data support to the early prevention, diagnosis and treatment of IS, and contribute to the development of IS targeted therapy strategies.

## Methods

### Study participants

The research subjects included 677 patients with IS in the acute phase (within 24 h of onset) and 681 normal individuals, who were recruited from the Affiliated Hospital of Northwest University (Xi'an No.3 Hospital). The inclusion criteria of the IS case group were to use the National Institutes of Health Stroke Scale (NIHSS) to assess the degree of neurological deficit, and to confirm the diagnosis by neurological examination, brain computed tomography (CT) and magnetic resonance imaging (MRI) (16). Patients with tumors, brain trauma, cerebral hemorrhage and cerebrovascular malformations were excluded. The control group was from patients who received physical examinations in the hospital during the same period without any family history of stroke, hypertension, diabetes and cardiovascular disease (3, 17). Trained staff administered questionnaires to participants to collect demographic data (including age, sex, smoking and drinking habits, and disease history), while collecting venous blood samples from recruiters who had fasted for at least 8 h. The protocol was approved by the Ethics Committee of this hospital and complies with the Declaration of Helsinki. Moreover, all participants signed an informed consent form.

### SNPs selection and genotyping

Three SNPs in *MMP2* (rs1053605, rs243849, and rs14070) were selected to identify *MMP2* candidate SNP variants based on NCBI database (https://www.ncbi.nlm.nih.gov/snp), minor allele frequency (MAF) >5%, and SNP was better assessed in previous studies (9, 18–20). After the extraction and purification of the above-mentioned sample DNA, primers for candidate SNPs (rs1053605, rs243849 and rs14070) were designed based on the gene sequence of *MMP2* using Primer5.0 primer design software (Supplementary Table 1). AgenaMassArray and AgenaTyper 4.0 were applied for genotyping and data analysis, respectively. Five percent of DNA samples were chosen for repeat testing to control quality, with >99% concordance of typing.

### Statistical analysis

The χ^2^ test and t test were used to perform statistical data processing analysis on demographic characteristics, allele and genotype frequencies of cases and controls. The Hardy-Weinberg Equilibrium (HWE) method was applied to assess the overall representativeness of the sample. Multivariate logistic regression was applied to calculate odds ratios (ORs) and 95% confidence intervals (CIs) after stratification for sex, age, smoking, and drinking. The associations between SNPs and IS risk in various models (allele, co-dominant, dominant, recessive and additive) and the degree of genetic association among the three SNPs were assessed using Plink software (version 1.9). In addition, multivariate dimensionality reduction (MDR) software was used to analyze the interactions among SNPs. In this study, *p* < 0.05 indicated a statistically significant difference. STRING and Oebiotech databases were adopted to analyze the biological activity and function analysis of *MMP2*.

## Results

### Participant characteristics

As depicted in Table 1, clinical characteristics of 677 IS patients and 681 normal control individuals included in this study. Notably, the mean age of the cases and the controls were 54.92 ± 6.79 and 55.64 ± 9.12, respectively, and there was no significant difference in age between the two groups (*P* = 0.099). In the case group, there were 455 males (67.2%) and 222 females (32.8%) with a sex ratio of 2.05:1. The sex ratio of 446 males (65.5%) and 235 females (34.5%) in the control group was 1.90:1. Thus, there was no obvious difference in gender distribution between the two groups (*P* = 0.528). Moreover, the distributions of smoking and drinking were not significantly different between cases and controls, both *P* = 0.704. In summary, there was no significant difference in clinical characteristics between the case and control groups, excluding the interference of confounding factors.

**Table 1:** Characteristics of ischemic stroke patients and controls.

**Variables**	**Cases (*****N*** = **677)**	**Controls (*****N*** = **681)**	***P*** **value**
Age, yr.	54.92 ± 6.79	55.64 ± 9.12	0.099^a^
Sex			0.528^b^
Male	455 (67.2%)	446 (65.5%)	
Female	222 (32.8%)	235 (34.5%)	
Smoking			0.704^b^
Yes	321 (47.4%)	330 (48.5%)	
No	356 (52.6%)	351 (51.5%)	
Drinking			0.704^b^
Yes	326 (48.2%)	335 (49.2%)	
No	351 (51.8%)	346 (50.8%)	

^a^P values were calculated from independent sample t-test.

^b^P values were calculated from two-sided χ^2^ test.

P < 0.05 indicates statistical significance.

### Candidate SNPs information and overall susceptibility

**Figure 1:**
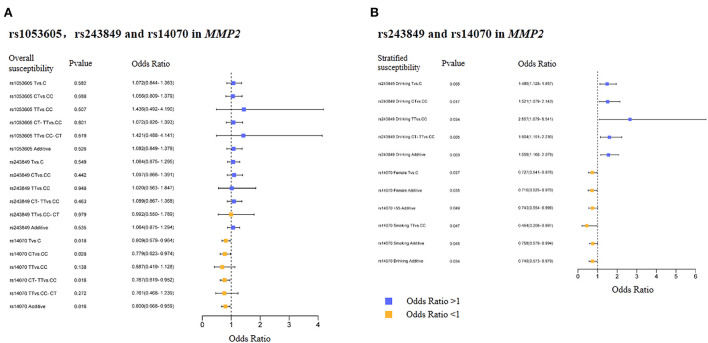
Overall and stratified analysis results of rs1053605, rs243849 and rs14070 in *MMP2*. **(A)** Overall results of rs1053605, rs243849, and rs14070 in *MMP2*. **(B)** Stratified significant results of rs243849, and rs14070 in *MMP2*.

The basic biological information of 3 SNPs (rs1053605, rs243849 and rs14070) in *MMP2* was presented in Table 2, which included chromosomes, physical location, function, MAF and HWE. The three SNPs in *MMP2* are located on chromosome 16. Both the MAF of cases and controls and the HWE of cases were >0.05, indicating that the selected samples were representative. Overall correlation analysis demonstrated that rs14070 in *MMP2* evidently reduced the risk of IS in multiple genetic models (Allele model: OR = 0.809, 95% CI = 0.679–0.964, *P* = 0.018; Co-dominant model: OR = 0.779, 95% CI = 0.623–0.974, *P* = 0.028; Dominance model: OR = 0.767, 95% CI = 0.619–0.952, *P* = 0.016; Log-additive model: OR = 0.800, 95% CI = 0.668–0.959, *P* = 0.016) (Table 3 and Figure 1A). However, rs1053605 and rs243849 in *MMP2* in Chinese Shaanxi population did not show an evident association with IS susceptibility in the overall analysis.

**Table 2:** Basic information of SNPs in *MMP2* gene.

**Gene**	**SNP**	**Chromosome**	**Position**	**Function**	**Allele (minor/ major)**	**MAF in** **Case**	**MAF in Control**	**HWE** ***P***
*MMP2*	rs1053605	16	55485695	Synonymous	T/C	0.114	0.107	0.553
*MMP2*	rs243849	16	55489793	Synonymous	T/C	0.184	0.175	0.595
*MMP2*	rs14070	16	55502815	Synonymous	T/C	0.226	0.265	0.140

SNP, single nucleotide polymorphism; MAF, minor allele frequency; HWE, Hardy-Weinberg equilibrium.

**Table 3:** Associations between SNPs of *MMP2* and ischemic stroke.

**SNP**	**Model**	**Genotype**	**Without adjusted**		**With adjusted**	
			**OR (95% CI)**	*P* ^1^	**OR (95% CI)**	*P* ^2^
rs1053605	Allele	C			1.000	
		T			1.072 (0.844–1.363)	0.582
	Co-dominant	CC	1.000		1.000	
		CT	1.053 (0.807–1.374)	0.703	1.056 (0.809–1.379)	0.688
		TT	1.364 (0.470–3.957)	0.568	1.436 (0.492–4.190)	0.507
	Dominant	CC	1.000		1.000	
		CT-TT	1.067 (0.822–1.385)	0.629	1.072 (0.826–1.393)	0.601
	Recessive	CC-CT	1.000		1.000	
		TT	1.349 (0.466–3.910)	0.581	1.421 (0.488–4.141)	0.519
	Log-additive	/	1.074 (0.843–1.369)	0.564	1.082 (0.849–1.379)	0.526
rs243849	Allele	C			1.000	
		T			1.064 (0.875–1.295)	0.549
	Co-dominant	CC	1.000		1.000	
		CT	1.093 (0.863–1.383)	0.463	1.097 (0.866–1.391)	0.442
		TT	1.033 (0.572–1.868)	0.914	1.020 (0.563–1.847)	0.948
	Dominant	CC	1.000		1.00	
		CT-TT	1.086 (0.865–1.363)	0.476	1.089 (0.867–1.368)	0.463
	Recessive	CC-TC	1.000		1.000	
		TT	1.006 (0.559–1.811)	0.984	0.992 (0.550–1.789)	0.979
	Log-additive	/	1.064 (0.875–1.293)	0.536	1.064 (0.875–1.294)	0.535
rs14070	Allele	C			1.000	
		T			0.809 (0.679–0.964)	0.018*
	Co-dominant	CC	1.000		1.000	
		CT	0.785 (0.628–0.981)	0.033*	0.779 (0.623–0.974)	0.028*
		TT	0.675 (0.412–1.107)	0.119	0.687 (0.419–1.128)	0.138
	Dominant	CC	1.000		1.000	
		CT-TT	0.771 (0.622–0.956)	0.018*	0.767 (0.619–0.952)	0.016*
	Recessive	CC-TC	1.000		1.000	
		TT	0.745 (0.459–1.212)	0.236	0.761 (0.468–1.239)	0.272
	Log-additive	/	0.800 (0.669–0.959)	0.016*	0.800 (0.668–0.959)	0.016*

SNP, single nucleotide polymorphism; OR, odds ratio; CI, confidence interval.

P values were calculated by logistic regression analysis, with P^1^ and P^2^ as without adjusted and with adjusted values, respectively.

Bold text and ^*^P < 0.05 represent statistical significance.

### Stratified analysis

In order to exclude the interference of environmental factors on the reliability of the results, we stratified the recruited samples by gender, age, smoking and drinking (Tables 4, 5 and Supplementary Table 2), with the results of significant correlation shown in Figure 1B. Statistical analysis results revealed that rs243849 in *MMP2* was significantly associated with the occurrence and development of IS risk under multiple genetic models of drinkers (Allele model: OR = 1.486, 95% CI = 1.128–1.957, *P* = 0.005; Co-dominant model: OR = 1.521, 95% CI = 1.079–2.143, *P* = 0.017; Dominance model: OR = 1.604, 95% CI = 1.151–2.236, *P* = 0.005; Log-additive model: OR = 1.558, 95% CI = 1.168–2.079, *P* = 0.003). Stratified results suggested that rs14070 in *MMP2* apparently reduced susceptibility to IS in additive model across females (OR = 0.716, 95% CI = 0.525–0.976, *P* = 0.035), participants with aged >55 (OR = 0.743, 95% CI = 0.554–0.998, P = 0.049), smoker (OR = 0.758, 95% CI = 0.579–0.994, *P* = 0.045) and drinker (OR = 0.749, 95% CI = 0.573–0.979, *P* = 0.034). In addition, the allelic model of rs14070 in *MMP2* evidently reduced the risk of IS in the female population (OR = 0.727, 95% CI = 0.541–0.976, P = 0.037) and the co-dominant model among smoking participants (OR = 0.454, 95% CI = 0.208–0.991, *P* = 0.047). Rs1053605 in *MMP2* was not associated with the risk of IS in multiple genetic models with different stratifications.

**Table 4:** Relationship between rs243849 of *MMP2* and ischemic stroke in different subgroups.

**Sex**										
**SNP**	**Model**	**Genotype**	**Male**				**Female**			
			**Case**	**Control**	**OR (95% CI)**	***P***	**Case**	**Control**	**OR (95% CI)**	***P***
rs243849	Allele	C	732	734	1.000		371	329	1.000	
		T	178	156	1.144 (0.902–1.452)	0.276	71	82	0.906 (0.639–1.283)	0.596
	Co-dominant	CC	305	292	1.000		160	158	1.000	
		CT	124	148	1.315 (0.980–1.764)	0.068	68	55	0.790 (0.513–1.216)	0.285
		TT	16	15	0.972 (0.466–2.029)	0.941	7	8	1.234 (0.421–3.622)	0.702
	Dominant	CC	305	292	1.000		160	158	1.000	
		CT-TT	140	163	1.274 (0.960–1.692)	0.093	75	63	0.829 (0.548–1.254)	0.374
	Recessive	CC-CT	429	440	1.000		228	213	1.000	
		TT	16	15	0.893 (0.431–1.852)	0.761	7	8	1.319 (0.453–3.842)	0.612
	Log-additive	/	/	/	1.181 (0.926–1.507)	0.180	/	/	0.898 (0.630–1.281)	0.553
**Age**										
**SNP**	**Model**	**Genotype**	**>55**				**≤55**			
			**Case**	**Control**	**OR (95% CI)**	***P***	**Case**	**Control**	**OR (95% CI)**	***P***
rs243849	Allele	C	628	488	1.000		475	634	1.000	
		T	132	110	0.933 (0.705–1.233)	0.668	117	128	1.220 (0.924–1.610)	0.176
	Co-dominant	CC	196	259	1.000		269	191	1.000	
		CT	96	110	0.936 (0.641–1.368)	0.733	96	93	1.372 (0.968–1.944)	0.076
		TT	7	11	1.051 (0.357–3.095)	0.928	16	12	0.989 (0.453–2.161)	0.979
	Dominant	CC	196	259	1.000		269	191	1.000	
		CT-TT	103	121	0.945 (0.654–1.366)	0.763	112	105	1.314 (0.943–1.831)	0.107
	Recessive	CC-CT	292	369	1.000		365	284	1.000	
		TT	7	11	1.073 (0.367–3.139)	0.898	16	12	0.902 (0.416–1.957)	0.794
	Log-additive	/	/	/	0.963 (0.696–1.333	0.821	/	/	1.191 (0.904–1.569)	0.214
**Smoking**										
**SNP**	**Model**	**Genotype**	**Smoking**				**Non-smoking**			
			**Case**	**Control**	**OR (95% CI)**	***P***	**Case**	**Control**	**OR (95% CI)**	***P***
rs243849	Allele	C	513	547	1.000		590	575	1.000	
		T	127	113	1.198 (0.905–1.587)	0.224	122	125	0.951 (0.723–1.252)	0.727
	Co-dominant	CC	230	206	1.000		235	244	1.000	
		CT	87	101	1.399 (0.983–1.990)	0.062	105	102	0.893 (0.638–1.249)	0.508
		TT	13	13	1.240 (0.551–2.790)	0.603	10	10	1.057 (0.422–2.651)	0.905
	Dominant	CC	230	206	1.000		235	244	1.000	
		CT-TT	100	114	1.379 (0.984–1.932)	0.062	115	112	0.906 (0.655–1.255)	0.554
	Recessive	CC-CT	317	307	1.000		340	346	1.000	
		TT	13	13	1.117 (0.500–2.492)	0.787	10	10	1.093 (0.438–2.725)	0.849
	Log-additive	/	/	/	1.272 (0.957–1.689)	0.097	/	/	0.935 (0.704–1.244)	0.646
**Drinking**										
**SNP**	**Model**	**Genotype**	**Drinking**				**Non-drinking**			
			**Case**	**Control**	**OR (95% CI)**	***P***	**Case**	**Control**	**OR (95% CI)**	***P***
rs243849	Allele	C	504	559	1.000		599	563	1.000	
		T	146	109	**1.486 (1.128–1.957)**	0.005**	103	129	0.751 (0.565–0.996)	0.052
	Co-dominant	CC	233	194	1.000		232	256	1.000	
		CT	93	116	**1.521 (1.079–2.143)**	0.017*	99	87	0.804 (0.567–1.140)	0.221
		TT	8	15	**2.657 (1.079–6.541)**	0.034*	15	8	0.455 (0.184–1.124)	0.088
	Dominant	CC	233	194	1.000		232	256	1.000	
		CT-TT	101	131	**1.604 (1.151–2.236)**	0.005**	114	95	0.757 (0.542–1.058)	0.103
	Recessive	CC-CT	326	310	1.000		331	343	1.000	
		TT	8	15	2.306 (0.945–5.633)	0.067	15	8	0.483 (0.196–1.186)	0.112
	Log-additive	/	/	/	**1.558 (1.168–2.079)**	0.003**	/	/	0.754 (0.566–1.005)	0.054

SNP, single nucleotide polymorphism; OR, odds ratio; CI, confidence interval.

P values were calculated by logistic regression analysis with adjusted.

Bold text and ^*^P < 0.05 or ^**^P < 0.01 represent statistical significance.

**Table 5:** Relationship between rs14070 of *MMP2* and ischemic stroke in different subgroups.

**Sex**										
**SNP**	**Model**	**Genotype**	**Male**				**Female**			
			**Case**	**Control**	**OR (95% CI)**	***P***	**Case**	**Control**	**OR (95% CI)**	***P***
rs14070	Allele	C	708	672	1.000		337	329	1.000	
		T	200	220	0.863 (0.693–1.074)	0.200	105	141	**0.727 (0.541–0.976)**	0.037*
	Co-dominant	CC	247	273	1.000		113	127	1.000	
		CT	178	162	0.788 (0.595–1.042)	0.095	103	83	0.747 (0.503–1.111)	0.150
		TT	21	19	0.827 (0.429–1.592)	0.569	19	11	0.468 (0.209–1.050)	0.066
	Dominant	CC	247	273	1.000		113	127	1.000	
		CT-TT	199	181	0.792 (0.604–1.038)	0.091	122	94	0.701 (0.479–1.026)	0.067
	Recessive	CC-CT	425	435	1.000		216	210	1.000	
		TT	21	19	0.908 (0.476–1.732)	0.769	19	11	0.531 (0.241–1.169)	0.116
	Log-additive	/	/	/	0.835 (0.663–1.051)	0.124	/	/	**0.716 (0.525–0.976)**	0.035*
Age										
**SNP**	**Model**	**Genotype**	**>** **55**				**≤55**			
			**Case**	**Control**	**OR (95% CI)**	***P***	**Case**	**Control**	**OR (95% CI)**	***P***
rs14070	Allele	C	589	441	1.000		456	560	1.000	
		T	169	157	0.806 (0.628–1.035)	0.096	136	204	0.819 (0.638–1.051)	0.130
	Co-dominant	CC	161	227	1.000		199	173	1.000	
		CT	119	135	0.732 (0.508–1.055)	0.095	162	110	0.790 (0.572–1.091)	0.152
		TT	19	17	0.575 (0.256–1.289)	0.179	21	13	0.763 (0.365–1.592)	0.471
	Dominant	CC	161	227	1.000		199	173	1.000	
		CT-TT	138	152	0.711 (0.500–1.012)	0.059	183	123	0.787 (0.576–1.075)	0.132
	Recessive	CC-CT	280	362	1.000		316	283	1.000	
		TT	19	17	0.650 (0.294–1.437)	0.287	21	13	0.840 (0.407–1.734)	0.638
	Log-additive	/	/	/	**0.743 (0.554–0.998)**	0.049*	/	/	0.824 (0.632–1.073)	0.150
**Smoking**										
**SNP**	**Model**	**Genotype**	**Smoking**				**Non-smoking**			
			**Case**	**Control**	**OR (95% CI)**	***P***	**Case**	**Control**	**OR (95% CI)**	***P***
rs14070	Allele	C	502	489	1.000		543	512	1.000	
		T	138	171	0.786 (0.608–1.016)	0.068	167	190	0.829 (0.652–1.054)	0.126
	Co-dominant	CC	180	193	1.000		180	207	1.000	
		CT	129	116	0.824 (0.592–1.147)	0.251	152	129	0.759 (0.553–1.041)	0.087
		TT	21	11	**0.454 (0.208–0.991)**	0.047*	19	19	1.006 (0.509–1.992)	0.985
	Dominant	CC	180	193	1.000		180	207	1.000	
		CT-TT	150	127	0.771 (0.560–1.062)	0.112	171	148	0.785 (0.579–1.064)	0.119
	Recessive	CC-CT	309	309	1.000		332	336	1.000	
		TT	21	11	0.491 (0.228–1.058)	0.069	19	19	1.129 (0.578–2.207)	0.723
	Log-additive	/	/	/	**0.758 (0.579–0.994)**	0.045*	/	/	0.860 (0.668–1.109)	0.245
**Drinking**										
**SNP**	**Model**	**Genotype**	**Drinking**				**Non-drinking**			
			**Case**	**Control**	**OR (95% CI)**	***P***	**Case**	**Control**	**OR (95% CI)**	***P***
rs14070	Allele	C	510	497	1.000		535	504	1.000	
		T	140	173	0.789 (0.611–1.017)	0.070	165	188	0.827 (0.649–1.053)	0.124
	Co-dominant	CC	182	199	1.000		178	201	1.000	
		CT	133	112	0.759 (0.545–1.057)	0.103	148	133	0.811 (0.590–1.114)	0.195
		TT	20	14	0.540 (0.256–1.140)	0.106	20	16	0.725 (0.358–1.468)	0.372
	Dominant	CC	182	199	1.000		178	201	1.000	
		CT-TT	153	126	0.730 (0.530–1.004)	0.053	168	149	0.800 (0.589–1.088)	0.155
	Recessive	CC-CT	315	311	1.000		326	334	1.000	
		TT	20	14	0.602 (0.289–1.256)	0.176	20	16	0.792 (0.396–1.584)	0.509
	Log-additive	/	/	/	**0.749 (0.573–0.979)**	0.034*	/	/	0.828 (0.640–1.071)	0.151

SNP, single nucleotide polymorphism; OR, odds ratio; CI, confidence interval.

P values were calculated by logistic regression analysis with adjusted.

Bold text and ^*^P < 0.05 represent statistical significance.

### Haplotype and MDR analysis

**Figure 2:**
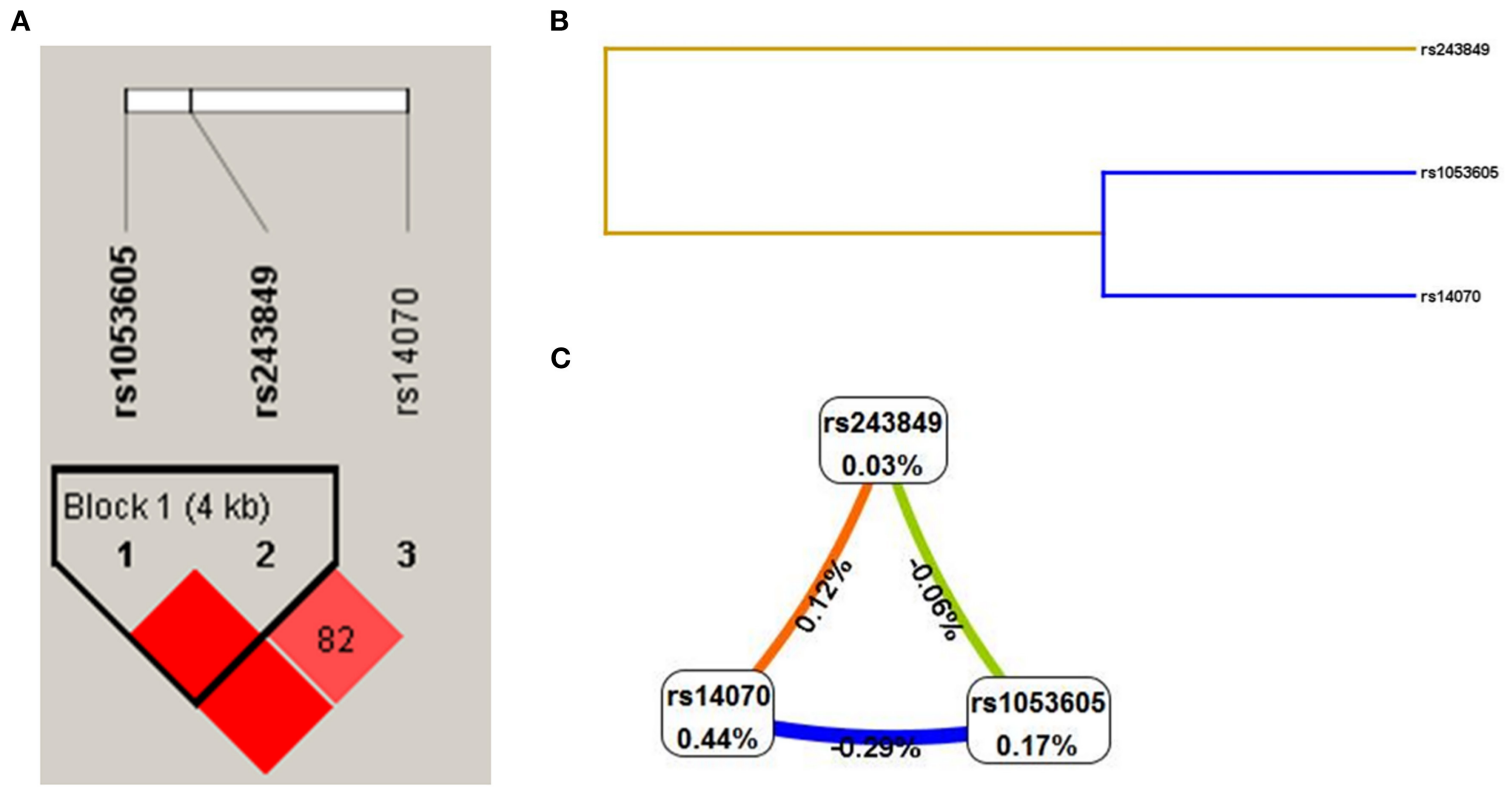
Genetic association between 3 SNPs (rs1053605, rs243849 and rs14070) in *MMP2*. **(A)** LD results of 3 SNPs in *MMP2*. **(B)** SNP-SNP interaction dendrogram of MDR analysis. **(C)** Fruchterman-reingold of MDR analysis (The closer to red the stronger the synergy, the closer to the blue the more redundancy).

The linkage disequilibrium (LD) results demonstrated that the three candidate SNPs (rs1053605, rs243849 and rs14070) of *MMP2* formed an LD block (Figure 2A), which illustrated that there was a strong linkage relationship among the SNPs of *MMP2*. Additionally, the results of MDR analysis claimed that the single-locus model (rs14070), the two-loci model (rs243849 and rs14070) and the three-loci model (rs1053605, rs243849 and rs14070) all had high test accuracy, high cross-validation consistency (CVC) and *P* < 0.05, as depicted in Table 6, Figures 2B,C. In conclusion, the three candidate SNPs in *MMP2* have strong genetic associations, and the interaction of gene polymorphisms may play an essential role in the genetic susceptibility of IS.

**Table 6:** *MMP2* SNP–SNP interaction models analyzed by the MDR method.

**Model**	**Training Bal. Acc**.	**Testing Bal. Acc**.	**CVC**	**OR (95% CI)**	***P***
rs14070	0.531	0.531	10/10	1.287 (1.038–1.596)	0.0214
rs243849, rs14070	0.544	0.530	10/10	1.457 (1.167–1.820)	<0.0001
rs1053605, rs243849, rs14070	0.554	0.529	10/10	1.572 (1.258–1.963)	<0.0001

### *MMP2* related functions

**Figure 3:**
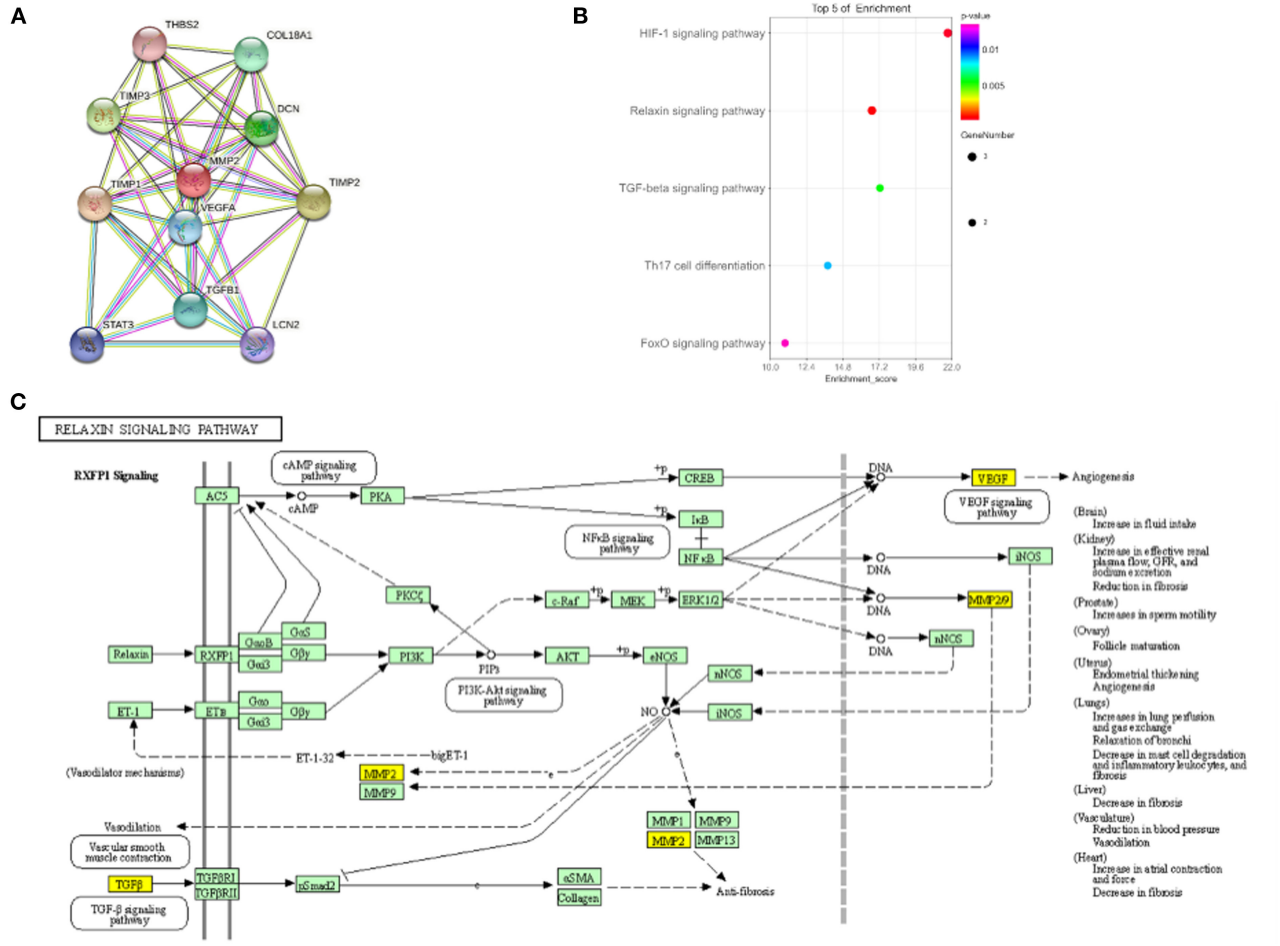
Biological function of *MMP2*. **(A)** Protein interaction diagram of *MMP2*. **(B)** KEGG enrichment results of *MMP2*. **(C)** *MMP2* involved in Relaxin signaling pathway.

To further explore the potential function of *MMP2* in regulating IS, protein-protein interaction figure and KEGG enrichment were constructed. It was indicated that *MMP2* can regulate the metalloproteinase inhibitor family (*TIMP1, TIMP2* and *TIMP3*) and angiogenesis-related targets such as *VEGFA* and *TGF-*β (Figure 3A). Pathway enrichment analysis demonstrated that *MMP2* and its related proteins mainly regulate relaxin signaling pathway involved in angiogenesis, inflammation and vascular endothelial function (Figures 3B,C). In brief, *MMP2* mainly regulated angiogenesis and inflammation and plays a pivotal role in the occurrence, development and recovery of IS.

## Discussion

IS is the leading cause of disability and death worldwide (10), a complex neurological disorder involving multiple factors including genetics and environment (4). The International Stroke Genetics Consortium suggested that genetic factors may account for up to 50% of an individual's risk of stroke (10). Previous extensive GWAS analyses have implicated genetic factors as a major contributor to stroke occurrence (21). Twenty-two new stroke risk loci were found to be significantly associated with stroke subtypes and pleiotropic models by GWAS analysis of 520,000 subjects from multiple countries (22). Studies have shown that people with high genetic risk have a 35% higher risk of stroke compared with people with low genetic risk, with a hazard ratio of 1.35 (95% CI: 1.21–1.50, *P* < 0.001) (23). In many neurological diagnoses, significant individual differences in rehabilitation outcomes may be associated with genetic variation. For example, rs6265 (val^66^met) on brain-derived neurotrophic factor (*BDNF*) significantly affected neuroplasticity after stroke in Chinese, Iranian, Korean and East Asian populations (24). In a nutshell, genetic factors are the main contributors to the occurrence, development and recovery of stroke.

As one of the major constituent enzymes in the brain (25), *MMP2* is a crucial target in the *MMPs* family that regulates ECM degradation and blood-brain barrier disruption (26). MMP-2 was involved in the process of stroke injury in the early stage, and its activity and protein expression were obviously increased at this time (26). At the same time, Claudin-5 and occludin were degraded by *MMP2*, the BBB was destroyed and the size of cerebral infarction increased (27). *MMP2* can also promote endogenous repair, especially angiogenesis, cerebral blood flow reconstruction and repair of brain tissue damage in the recovery stage after stroke (25). In general, *MMP2* is an important regulatory indicator of stroke occurrence and recovery.

The regulatory role of *MMP2* may be related to the regulation of its genetic variation. Li et al. (13) illustrated that compared with the normal control group, the frequency of CC genotype and C allele of *MMP2* 735C/T in the first and recurrent IS in the Chinese population were significantly increased, and the frequency of IS recurrence was more significant. Previous studies demonstrated that rs243849 in *MMP2* evidently reduced the risk of IS in Hainan population, while rs1053605 in *MMP2* was not found to be associated with IS susceptibility (9). At present, no study has explored the correlation between rs14070 in *MMP2* and IS susceptibility, only found that rs14070 was positively correlated with the incidence of hypertension caused by urinary cadmium (20). In our study, the results showed that rs1053605 was not significantly associated with IS susceptibility in Chinese Shaanxi population, which was consistent with the results reported in the literature. This study indicated that rs243849 in *MMP2* obviously increase the risk of IS in drinkers under multiple genetic models, however, rs243849 in *MMP2* in the literature evidently reduced the risk of IS in Hainan population. Apart from drinking, the reasons for this difference may be related to environmental, climatic and dietary factors in the northern and southern Chinese populations. Furthermore, this study was the first to confirm that rs14070 in *MMP2* apparently reduced the risk of IS in Chinese Shaanxi population under multiple genetic models. In brief, the polymorphism differences of *MMP2* were significantly associated with the risk of IS in Chinese Shaanxi populations, and the key role of *MMP2* may depend on polymorphism differences.

Genetic susceptibility to IS may be associated with race, age, smoking, and drinking. A study found that long-term smoking, body mass index (BMI) ≥30, inactivity or unhealthy diet will increase the risk of IS by 66% (23). Rs1800795 in *IL-6* in Asian populations was significantly associated with stroke occurrence, whereas rs1800795 on *IL-6* in Oceania populations was not associated with IS occurrence (1). Another study demonstrated that SNPs in *PITX2* significantly reduced the risk of IS in Chinese Han males (2). In addition, SNPs in *HTRA1* were significantly associated with the risk of IS among Chinese Han smokers (5). In our study, analysis results illustrated that rs243849 in *MMP2* was associated with evidently increased IS risk in Chinese drinking population under allelic, co-dominant, dominant and additive genetic models. However, rs14070 in *MMP2* can still significantly reduce IS susceptibility in Chinese Shaanxi population older than 55 years, females, smokers and drinkers. These study suggested that genetic susceptibility to IS is closely related to race, sex, advanced age, smoking and drinking.

The function of *MMP2* depends on endogenous inhibitors (*TIMP*), angiogenesis factors (*VEGF, TGF-*β) and inflammatory factors (28). The regulation of ECM degradation mainly depends on the balance between MMPs-TIMP (29). Imbalance of *MMP*-*TIMP* can lead to neurological diseases (stroke, Alzheimer's disease), atherosclerosis and cardiovascular diseases (30). Notably, the secretion and regulation of *MMP2* lead to BBB damage in the early stage of stroke (31). As a major inhibitor of *MMP2* (19), *TIMP2* likewise has a dual role (29). *VEGF* interacting with *MMP2* regulated neovascular remodeling and neuroprotection after stroke injury (32). Similarly, *TGF-*β can promote the remodeling of ECM and inhibit the disruption of BBB (32). Furthermore, *MMP-2* and *TGF-*β can be bidirectionally regulated, which is beneficial to angiogenesis and reconstruction (30). In this study, we demonstrated that *MMP2* mainly regulated *TIMPs* and angiogenesis factors (*VEGF, TGF-*β). Subsequently, they participated in the Relaxin signaling pathway to regulate angiogenesis, inflammation and vascular endothelial function. Collectively, *MMP2* can regulate inflammation, angiogenesis, and ECM degradation, which play a crucial role in IS occurrence and recovery.

Interesting findings were revealed in this study, which provided a reliable basis to future research on *MMP2* regulation of IS. However, potential limitations of our study deserve consideration. In this study, only 3 SNPs in *MMP2* were explored, and we will continue to explore the association of other SNPs in *MMP2*, MMPs and gene interactions with IS susceptibility in the future (16). Additionally, the current study was limited to a single ethnic group, other ethnic groups should be validated in the future (13, 17). Finally, in order to further determine the potential impact of *MMP2* genetic variation on the risk of IS, cell and animal models will be required to verify its regulatory mechanism in different stages of IS (2).

## Conclusion

In general, this study explored the association of rs1053605, rs243849 and rs14070 in *MMP2* with the risk of IS in Chinese Shaanxi population. Stratified analysis indicated that rs243849 in *MMP2* obviously increased the risk of IS among drinking population, while rs14070 in *MMP2* evidently reduced IS susceptibility in females, participants with older than 55, smokers, and drinkers. This study illustrated that genetic variation of *MMP2* played an essential role in the occurrence and recovery of IS, which provided support for the early diagnosis and treatment of IS.

## Data availability statement

The datasets generated and/or analyzed during the current study are available in the zenodo repository (https://zenodo.org/record/6826121#.Ys6AkfkaWUk).

## Ethics statement

The studies involving human participants were reviewed and approved by this study was performed in line with the principles of the Declaration of Helsinki. Approval was granted by the Ethics Committee of the Affiliated Hospital of Northwest University (Xi'an No.3 Hospital). The patients/participants provided their written informed consent to participate in this study.

## Author contributions

SL and SY performed the manuscript. XiaobZ and YZ took part in genotyping. JZ, XiaoZ, and WL participated in the statistical analysis of the data. XN and GZ modified the manuscript. WS, MC, and YT designed the study, co-supervised the work, and finalized the manuscript. All authors have read and approved the final manuscript.

## Funding

This study was supported by Natural Science Foundation of China (No. 82104155), Key Research and Development Program of Shaanxi (2020ZDLSF04-03 and 2021SF-096), and Xi'an Science and Technology Planning Project (21YXYY0038 and 21YXYJ0004).

## Conflict of interest

The authors declare that the research was conducted in the absence of any commercial or financial relationships that could be construed as a potential conflict of interest.

## Publisher's note

All claims expressed in this article are solely those of the authors and do not necessarily represent those of their affiliated organizations, or those of the publisher, the editors and the reviewers. Any product that may be evaluated in this article, or claim that may be made by its manufacturer, is not guaranteed or endorsed by the publisher.
