# Supplementary material for: Impact of MMP2 rs243849 and rs14070 genetic polymorphisms on the ischemic stroke susceptibility in Chinese Shaanxi population
Source: Front Neurol. 2022 Jul 25;13:931437. doi: 10.3389/fneur.2022.931437 (PMC9358222; doi:10.3389/fneur.2022.931437)
Supplement: Supplementary file 1 [file Table_1.DOCX]

**Supplementary table 1 Primer sequence of rs1053605, rs243849 and rs14070 in *MMP2***

| SNP | 1^st^-PCRP | 2^nd^-PCRP | UEP_SEQ |
| --- | --- | --- | --- |
| rs1053605 | ACGTTGGATGCTCAAAGTTGTAGGTGGTGG | ACGTTGGATGAAGGAGTACAACAGCTGCAC | ACAGCTGCACTGATAC |
| rs243849 | ACGTTGGATGTTCCAGGCATCTGCGATGAG | ACGTTGGATGAGTGACGGAAAGATGTGGTG | AAATGGATCCTGGCTT |
| rs14070 | ACGTTGGATGTACCTTGGTCAGGGCAGAAG | ACGTTGGATGGAAGTGTCCTTTAGAGAGGC | tgttCGACCACAGCCAACTACGATGA |

**Supplementary table 2 Relationship between rs1053605 of *MMP2* and stroke in different subgroups**

| Sex |  |  |  |  |  |  |  |  |  |  |
| --- | --- | --- | --- | --- | --- | --- | --- | --- | --- | --- |
| SNP | Model | Genotype | Male | | | | Female | | | |
|  |  |  | Case | Control | OR (95% CI) | *P* | Case | Control | OR (95% CI) | *P* |
| rs1053605 | Allele | C | 810 | 794 | 1.000 |  | 386 | 422 | 1.000 |  |
|  |  | T | 98 | 98 | 0.980 (0.729-1.319) | 0.940 | 56 | 48 | 1.275 (0.847-1.921) | 0.253 |
|  | Co-dominant | CC | 353 | 358 | 1.000 |  | 188 | 171 | 1.000 |  |
|  |  | CT | 88 | 94 | 1.078 (0.774-1.503) | 0.656 | 46 | 44 | 1.133 (0.704-1.824) | 0.607 |
|  |  | TT | 5 | 2 | 0.547 (0.104-2.889) | 0.478 | 1 | 6 | 5.907 (0.674-51.75) | 0.109 |
|  | Dominant | CC | 353 | 358 | 1.000 |  | 188 | 171 | 1.000 |  |
|  |  | CT-TT | 93 | 96 | 1.053 (0.760-1.461) | 0.755 | 47 | 50 | 1.242 (0.782-1.973) | 0.359 |
|  | Recessive | CC-CT | 441 | 452 | 1.000 |  | 234 | 215 | 1.000 |  |
|  |  | TT | 5 | 2 | 0.539 (0.102-2.842) | 0.466 | 1 | 6 | 5.759 (0.659-50.31) | 0.113 |
|  | Log-additive | / | / | / | 1.023 (0.751-1.393) | 0.885 | / | / | 1.321 (0.867-2.013) | 0.196 |
| Age |  |  |  |  |  |  |  |  |  |  |
| SNP | Model | Genotype | > 55 | | | | ≤ 55 | | | |
|  |  |  | Case | Control | OR (95% CI) | *P* | Case | Control | OR (95% CI) | *P* |
| rs1053605 | Allele | C | 669 | 521 | 1.000 |  | 527 | 695 | 1.000 |  |
|  |  | T | 91 | 77 | 0.920 (0.665-1.273) | 0.620 | 63 | 69 | 1.204 (0.840-1.726) | 0.311 |
|  | Co-dominant | CC | 226 | 293 | 1.000 |  | 315 | 236 | 1.000 |  |
|  |  | CT | 69 | 83 | 0.770 (0.512-1.158) | 0.209 | 65 | 55 | 1.175 (0.784-1.762) | 0.435 |
|  |  | TT | 4 | 4 | 0.483 (0.112-2.093) | 0.331 | 2 | 4 | 3.383 (0.592-19.34) | 0.171 |
|  | Dominant | CC | 226 | 293 | 1.000 |  | 315 | 236 | 1.000 |  |
|  |  | CT-TT | 73 | 87 | 0.750 (0.503-1.117) | 0.157 | 67 | 59 | 1.233 (0.829-1.834) | 0.300 |
|  | Recessive | CC-CT | 295 | 376 | 1.000 |  | 380 | 291 | 1.000 |  |
|  |  | TT | 69 | 83 | 0.512 (0.119-2.211) | 0.370 | 2 | 4 | 3.282 (0.575-18.73) | 0.181 |
|  | Log-additive | / | / | / | 0.753 (0.522-1.085) | 0.128 | / | / | 1.272 (0.879-1.841) | 0.202 |
| Smoking |  |  |  |  |  |  |  |  |  |  |
| SNP | Model | Genotype | Smoking | | | | Non-smoking | | | |
|  |  |  | Case | Control | OR (95% CI) | *P* | Case | Control | OR (95% CI) | *P* |
| rs1053605 | Allele | C | 559 | 591 | 1.000 |  | 637 | 625 | 1.000 |  |
|  |  | T | 81 | 69 | 1.241 (0.882-1.746) | 0.225 | 73 | 77 | 0.930 (0.663-1.305) | 0.730 |
|  | Co-dominant | CC | 267 | 244 | 1.000 |  | 274 | 285 | 1.000 |  |
|  |  | CT | 57 | 71 | 1.312 (0.879-1.959) | 0.184 | 77 | 67 | 0.849 (0.584-1.236) | 0.393 |
|  |  | TT | 6 | 5 | 0.988 (0.287-3.405) | 0.985 | 0 | 3 | - | - |
|  | Dominant | CC | 267 | 244 | 1.000 |  | 274 | 285 | 1.000 |  |
|  |  | CT-TT | 63 | 76 | 1.283 (0.871-1.889) | 0.208 | 77 | 70 | 0.888 (0.612-1.287) | 0.530 |
|  | Recessive | CC-CT | 324 | 315 | 1.000 |  | 351 | 352 | 1.000 |  |
|  |  | TT | 6 | 5 | 0.940 (0.273-3.230) | 0.921 | 0 | 3 | - | - |
|  | Log-additive | / | / | / | 1.212 (0.859-1.711) | 0.274 | / | / | 0.939 (0.655-1.346) | 0.732 |
| Drinking |  |  |  |  |  |  |  |  |  |  |
| SNP | Model | Genotype | Drinking | | | | Non-drinking | | | |
|  |  |  | Case | Control | OR (95% CI) | *P* | Case | Control | OR (95% CI) | *P* |
| rs1053605 | Allele | C | 579 | 599 | 1.000 |  | 617 | 617 | 1.000 |  |
|  |  | T | 71 | 71 | 1.035 (0.730-1.465) | 0.859 | 83 | 75 | 1.107 (0.794-1.542) | 0.556 |
|  | Co-dominant | CC | 269 | 260 | 1.000 |  | 272 | 269 | 1.000 |  |
|  |  | CT | 61 | 59 | 1.000 (0.665-1.506) | 0.998 | 73 | 79 | 1.083 (0.748-1.566) | 0.674 |
|  |  | TT | 5 | 6 | 1.457 (0.427-4.976) | 0.548 | 1 | 2 | 2.062 (0.177-24.01) | 0.563 |
|  | Dominant | CC | 269 | 260 | 1.000 |  | 272 | 269 | 1.000 |  |
|  |  | CT-TT | 66 | 65 | 1.033 (0.696-1.532) | 0.872 | 74 | 81 | 1.096 (0.760-1.580) | 0.625 |
|  | Recessive | CC-CT | 330 | 319 | 1.000 |  | 345 | 348 | 1.000 |  |
|  |  | TT | 5 | 6 | 1.457 (0.428-4.965) | 0.547 | 1 | 2 | 2.026 (0.174-23.56) | 0.573 |
|  | Log-additive | / | / | / | 1.058 (0.746-1.500) | 0.753 | / | / | 1.107 (0.776-1.580) | 0.574 |

SNP: single nucleotide polymorphism; OR: odds ratio; CI: confidence interval.

*P* values were calculated by logistic regression analysis with adjusted.
